# The Effect of a Diverse Dataset for Transfer Learning in Thermal Person Detection

**DOI:** 10.3390/s20071982

**Published:** 2020-04-02

**Authors:** Noor Ul Huda, Bolette D. Hansen, Rikke Gade, Thomas B. Moeslund

**Affiliations:** Visual Analysis of People Lab, Aalborg University, Rendsburggade 14, 9000 Aalborg, Denmark; bdha@create.aau.dk (B.D.H.); rg@create.aau.dk (R.G.); tbm@create.aau.dk (T.B.M.)

**Keywords:** thermal, person, databases, deep learning, CNN, images, detection, outdoor, dataset, model adaptation

## Abstract

Thermal cameras are popular in detection for their precision in surveillance in the dark and for privacy preservation. In the era of data driven problem solving approaches, manually finding and annotating a large amount of data is inefficient in terms of cost and effort. With the introduction of transfer learning, rather than having large datasets, a dataset covering all characteristics and aspects of the target place is more important. In this work, we studied a large thermal dataset recorded for 20 weeks and identified nine phenomena in it. Moreover, we investigated the impact of each phenomenon for model adaptation in transfer learning. Each phenomenon was investigated separately and in combination. the performance was analyzed by computing the F1 score, precision, recall, true negative rate, and false negative rate. Furthermore, to underline our investigation, the trained model with our dataset was further tested on publicly available datasets, and encouraging results were obtained. Finally, our dataset was also made publicly available.

## 1. Introduction

Person detection is the backbone of many applications ranging from surveillance and military to traffic analysis. Many computer vision branches like behavior analysis, activity recognition, threat recognition, and person re-identification start with the challenge of person detection.

**Figure 1:**
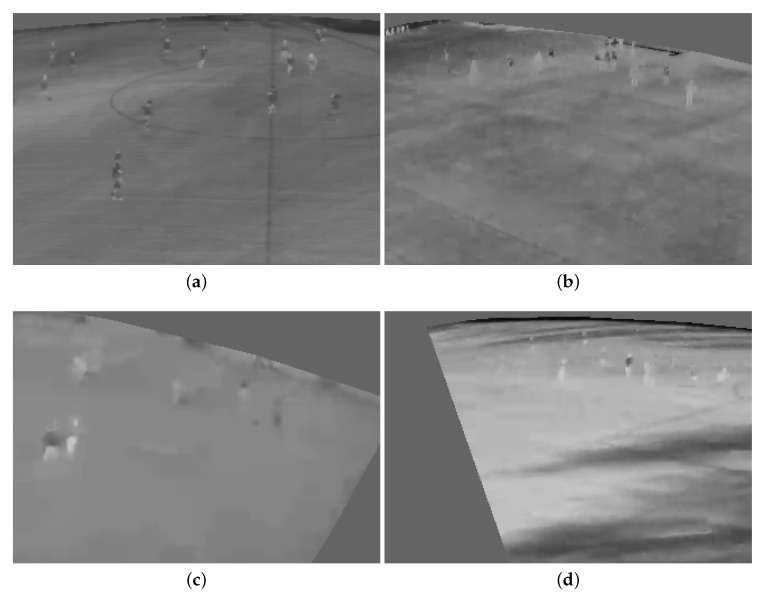
Some challenging characteristics in thermal data. (**a**) Varying body temperatures. (**b**) Similar temperatures. (**c**) Motion blur due to wind. (**d**) Shadows.

Visual cameras capturing visible light, as well as thermal cameras capturing infrared radiation have been utilized for person detection. Many feature based machine learning [1,2,3,4], as well as deep learning [5,6,7] approaches have been utilized to deal with the problem of person detection in thermal images. Even though thermal cameras have an advantage in outdoor person detection, due to the independence of illumination, robust detection still becomes very challenging in diverse weather and light conditions (see Figure 1) and is therefore far from a solved problem.

In the last decade, many deep learning based networks [8,9,10,11,12,13,14] have been abundantly created and utilized for person detection in color images. The key to success in the area of machine learning and deep learning is the availability of many datasets [1,13,14,15,16]. Recording and processing of large amount of dataset take much effort and many resources. Alternatively, currently, single shot detectors [8,10,11] and transfer learning are also gaining the attention of developers due to their speedy detection and fewer data requirements. Transfer learning refers to learning for a task by transferring the knowledge from the learning of another task. In deep learning, it refers to a method where a model for one task is reused as a starting point for training another task [17]. This reduces the data required, as well as the time needed for training. While learning based approaches have been successful in many computer vision and data domains, there is still a large gap in being able to solve thermal detection and classification problems due to the lack of a comprehensive and diverse dataset.

We reviewed the thermal datasets that are available publicly and can be used for person detection. Most of the publicly available thermal datasets (see Table 1) are either for tracking or classification. They are short sequences with little variability in the scene, i.e., weather conditions, light conditions, and person heat radiation. This drawback decreases the generalization of detectors. Furthermore, most of the thermal datasets available for person detection are pedestrian data from traffic scenarios and captured from the front view, which makes it difficult to detect people far from the camera. Only one dataset is available that has weather information including haze, rain, and cloudy conditions [18]. However, it contains only a small number of images and hence fails to generalize.

Capturing and annotating a large amount of thermal data are still challenging. An optimal solution would be to study a large range of data and utilize the tool of transfer learning to learn from RGB data. A different range of phenomena affecting thermal videos in the outdoor environment have not been investigated and described yet. Observing the effect of various data phenomena from thousand of hours of video can help in optimizing dataset development and annotation. The study requires a large dataset recorded over several weeks in different positions and in different places to make sure that all possible outdoor phenomena are covered.

As our first contribution, we studied 20 weeks of variable outdoor thermal data thoroughly to find different phenomena that affect the images. Even by determining all the phenomena, it is still questionable what kind of data are going to have a positive effect and which kind will have a negative effect on person detection in outdoor environments while training a network. Generally, it is presumed that the higher the number of images, the better the detection results. However, due to the high variation of the data characteristics and the low resolution of the thermal images, this is not necessarily the case here, as some phenomena might contribute to a high FP rate. To investigate this research question, as our second contribution, we categorized the phenomena and performed an ablation study for each category. This study gave us a deep analysis of the impact of each category of thermal data and let us choose data in an intelligent manner. This analysis was performed using a single shot deep network and the tool of transfer learning. We employed a single shot deep network due to its high performance and fast learning rate. Finally, the third contribution of this article was a new public thermal dataset for thermal person detection that contains variations regarding the time of day, weather, distance to the camera, various body vs. background temperatures, and shadows. The thermal weights will also be available for researchers for further utilization for transfer learning and solving other thermal data problems.

The rest of the paper is organized as follows: Section 2 provides an overview of the related work. In Section 3, we present our new dataset, and in Section 4, we conduct a thorough investigation into the role of novel training data in transfer learning. Finally, in Section 5, we discuss our findings and future perspectives.

## 2. Related Work

To create an understanding of thermal person detection, the following provides an overview of the state-of-the-art techniques, as well as the datasets used for the evaluation of these techniques.

### 2.1. Multimodal Approaches

Hwang et al. [1] presented a benchmark dataset and baseline code for detection of pedestrians in RGB-Thermal (RGB-T) data. Lahmayed et al. [19] presented a method based on multi-threshold and Histogram of Oriented Gradients (HOG) and Histograms of Oriented Optical Flow (HOOF) color features combined with an SVM using both thermal infrared and visible light images. They tested their algorithm on the OSU color thermal dataset [20],video analytic dataset [21], and LITIVdataset [22]. Fritz et al. [23] investigated the generalization of a deep learning network in multispectral person detection datasets. They mainly used the Caltech [24], city person [25], CVC-09 [26], KAIST [1], OSU color thermal [20], and Tokyo segmentation [27] datasets for their investigation. Li et al. [28] used the KAISTdataset [1] to create a person detector baseline and then narrowed it down by mining hard negatives. Cuerda et al. [29] employed stream selection based on the confidence map. In this way, they were able to choose the best image out of thermal and visible data based on day and night confidence maps. Many feature extraction and deep learning based approaches have been used for dealing with multimodal data. The problem with multimodal based techniques is the complexity in data handling, as well as the complexity in hardware installation. Here, we are more concerned about thermal only approaches.

### 2.2. Thermal Approaches

Thermal cameras have been utilized in many scenarios ranging from industry to daily life applications [30]. Much research has been carried out for person detection in the infrared domain. Dai et al. [31] presented a method based on background subtraction and shape based classification. They tested their method on the OSU thermal pedestrian database [18]. Zhang et al. [4] also presented a method based on background subtraction and boundary gradients, the temporal coherence of the object area, and the region signature of the intensity distribution. They also tested their method on the OSU thermal database [18]. Li et al. [2] implemented the pedestrian detection in infrared imagery by tuning HOG features. They also tested their algorithm on the OSU thermal pedestrian dataset [18]. A two-stage person recognition approach based on Maximally Stable Extreme Regions (MSERs) and verification of the detected hot spots using a Discrete Cosine Transform (DCT) based descriptor was proposed by Teutsch et al. [3]. They evaluated their approach on the OSU thermal pedestrian [18], OSU color thermal [20], and Terravic motion IR datasets [32]. Many [29,33,34,35,36,37,38,39] used their own datasets for the evaluation.

Recently, Herrmann et al. [5] tested the Single Shot Detector (SSD) with different preprocessing methods to assess thermal performance. They used KAIST [1] for performance evaluation. They [5] also worked with MSERs and CNN and tested on the AMROS, OSU thermal pedestrian [18], OSU color thermal [20], and Terravic motion IR [32] datasets. Tumas et al. [6] proposed an HOG based pedestrian detector combined with CNN for the FIR domain. Heo et al. [7] proposed adaptive Boolean map based saliency combined with YOLO for pedestrian detection at night time. They used CVC-09 [26] for their experiments. For sports player detection, Gade et al. [37,38,40] presented a method based on background subtraction and automatic thresholding. They tested their method on the indoor thermal dataset [40]. Huda et al. [39] previously suggested a simulation based occlusion handling method for detecting and counting the players. This was tested on their own sports dataset.

### 2.3. Datasets

Different multimodal and thermal datasets are publicly available for traffic analysis, surveillance, person tracking, and human pose estimation, among others. The datasets that can be used for person detection are listed in Table 1. The scene characteristics, type of data, number of frames, viewpoint, and scene characteristics/or main purpose of the datasets are also provided in the table. All these datasets can be used as pre-training of another network according to the application area.

All the datasets available consisted of sequences with a short duration; thus, they had less variability in terms of weather and light conditions. Most of the available datasets were pedestrian data from traffic data analysis and captured from a frontal viewpoint. Many datasets were indoor, and thus, these were captured in controlled light and temperature conditions and did not include all the variability of outdoor environments. Even with a large number of frames [1] and weather information [18], it was still questionable if the data were enough to include all outdoor phenomena. Therefore, the research community lacks a comprehensive and diverse dataset to develop robust algorithms for the detection of people. Therefore, we studied long durations of data and came up with a shorter, but novel and diverse dataset below that is comprised of all outdoor phenomena.

## 3. Novel Dataset

The first contribution of this paper is the investigation and study of a diverse thermal dataset for person detection. In thermal images, weather conditions have a similar effect as lighting conditions have on RGB images. it is therefore essential to include varying weather and light effects in a dataset. Furthermore, because the resolution of thermal sensors is still relatively low, the size of objects in images is also an important factor. The data we recorded were captured in outdoor sports fields with people playing soccer or performing related exercises. The nature of these recordings ensured that many challenges related to person detection were included: different scales, pose variations, interactions/occlusions between people, and fast and erratic motion. Regarding the weather effects, we recorded 20 weeks of thermal recordings across January to April in Denmark. Therefore, it spanned the periods from little daylight to bright sunny days and snowy days of winter to pleasant spring days. In the recordings, we experienced several different key challenges: varying temperatures (people hotter/colder/same temperature than the ground), shadows (parts of the ground were not heated by the Sun), wind (camera moving), snow (regions on the ground with different reflection and emissivity of heat), and occlusion (people in groups) in the thermal images.

**Figure 2:**
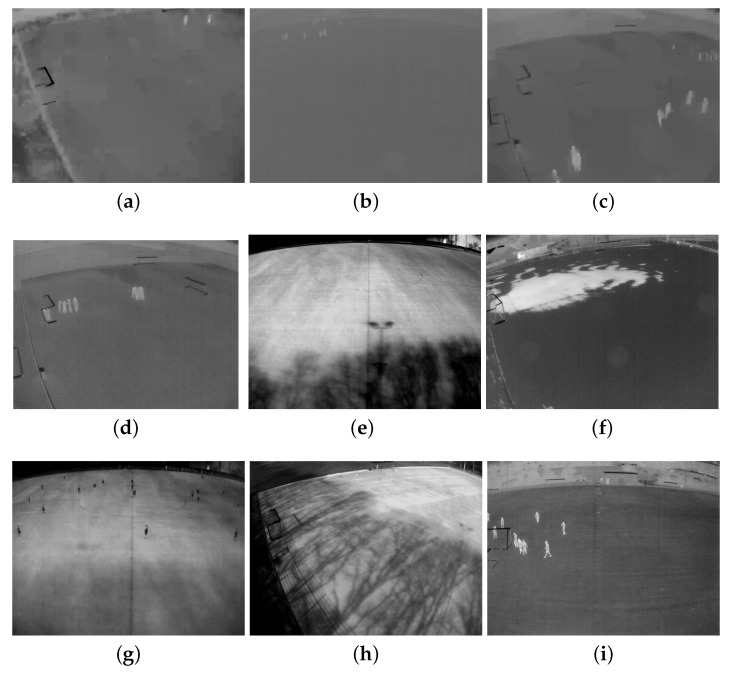
Nine different phenomena that are included in our novel dataset. (**a**) Low resolution. (**b**) Far viewpoint. (**c**) Wind. (**d**). Occlusion. (**e**) Shadow. (**f**) Snow. (**g**) Opposite temperature. (**h**) Similar temperature. (**i**) Good condition.

After examining all challenges and scrutinizing the entirety of the data, we suggested that nine different phenomena should be included in a dataset for it to be sufficiently diverse and help the model generalize outdoor person detection in thermal images. These nine phenomena are listed and illustrated in Figure 2.

### 3.1. Data Recording

We recorded thermal videos from 10 different sports fields for two weeks each, which comprised 20 weeks of data. The cameras used for recording were Axis Q1921 (resolution 384 × 288 pixels) and Axis 1922 (resolution 640 × 480), and they were mounted approximately 9m above the ground on a light pole surrounding the field. Three cameras were installed at the center of each field to cover the entire field area. The sequences selected for this investigation were from all of the cameras’ views. The recordings were done from January 2018 to April 2018.

### 3.2. Data Description

As the first step in transfer learning is a model adaptation, we used 3000 indoor publicly available images [40] as pre-training images for model adaptation. The dataset from [40] was selected for pre-training as it had nearly perfect thermal data, i.e., lighter person on a darker background. Moreover, it was similar to our dataset as it was recorded in an indoor sports field and contained 24,000 person annotations. As the data from [40] helped in model adaptation and saved in annotation cost, our new dataset (Table 2) helped in obtaining the goal of generalization in detection as it included all possible outdoor phenomena from an outdoor environment.

Manually annotating all the data was unrealistic. Therefore, we scrutinized the periods where all nine phenomena occurred, and the number of players in a given image in these periods varied (from 0 to 40). In each period, we selected a frame every 160th second and annotated that frame. This large temporal gap between annotated frames was introduced to enforce as much diversity as possible. One-thousand nine-hundred forty-one frames were selected as the training dataset. In these frames, a total of 5590 persons were annotated. The details of the dataset are presented in Table 2. For testing purposes, 1000 more frames were randomly selected from all the recorded data (100 frames from two weeks of video). it was manually checked that no image from the training data was repeated in the testing data. The camera view (left, right, middle) was also selected randomly. All of the data were annotated with the MATLAB object detection bounding box annotator [48]. Our person detection dataset (PD-T) is available at http://www.vap.aau.dk/dataset/.

## 4. Investigating the Role of Training Data

A traditional deep learning network contains a large number of parameters. Training such a network requires an enormous amount of training data. The online availability of such an enormous amount of data is not always a possibility, especially in non-RGB applications. Transfer learning is the optimal solution in such conditions since many features in the first layers of a deep learning network are similar across applications [49]. The question is which phenomena need to be included in a dataset for outdoor thermal person detection for a positive transfer. To investigate this research question, we needed a pre-trained detection algorithm on which we could apply transfer learning with our data. we chose the CNN based single shot detector YOLOv3 [8].

You Only Look Once (YOLO) is one of the fastest deep learning algorithms for the detection of objects in an image, which can process 45 frames per second. This algorithm treats the problem of detection as a regression problem and trains on the whole image at once to optimize the performance. Moreover, it detects the class objects with their probabilities at the same time without requiring region proposals.

The YOLOv3 network, used in this work, divided every training image into a grid of (S×S) cells. it searched for the center of the target objects in these grid cells. *B* number of bounding boxes with their confidence scores could be predicted by each grid cell. Confidence was defined as the probability of detected objects multiplied by the Intersection over Union (IoU) between the ground truth bounding box area and the detected object bounding box area.

The model was more effective at detecting small objects compared to previous versions of YOLO because it predicted bounding boxes at different scales. This added multiscale detection in v3 allowed us to detect a person very far from the camera. At the same time, the number of predictable bounding boxes in each cell provided some limitation on the detection.

### 4.1. Assessment Protocol

To assess the role of training data, we divided our training data based on the phenomena discussed in Section 3 into categories defined in Table 2. The amount of test data was always kept the same. Tests were performed by adding one category of images at a time and then combining different categories of images. A total of 16 different combinations were tested, listed in Table 3. Indoor data were from [40] and were used as a baseline for model adaptation. Results for each of these combinations would provide insights into how different types of training data affected the detection results on varying data.

For transfer learning, we used convolution weights that were pre-trained on ImageNet [14] using the Darknet53 [8] model due to their reported high performance and speed [8]. The network was trained with S = 7, where network iterations were set to 40,000, and the results from the mean of iterations (10,000, 20,000, 30,000, and 40,000) were considered. Here, we set the learning rate to 0.001, momentum to 0.9, and decay to 0.0005. The training and testing of all combinations were performed using a graphical processing unit GTX 1080 with Linux Ubuntu 16.04.

### 4.2. Evaluation

**Figure 3:**
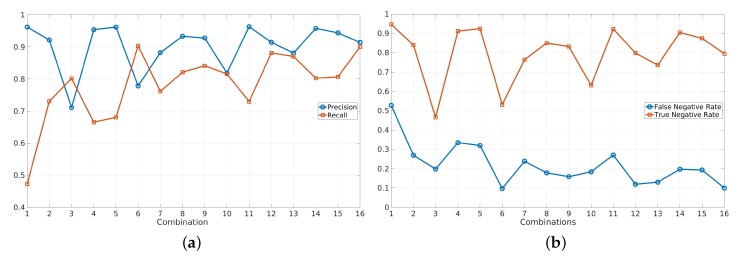
(**a**) Precision and Recall, (**b**) True Negative Rate (TNR) and False Negative Rate (FNR).

We used precision, recall, F1 score, False Negative Rate (FNR), and True Negative Rate (TNR) as the performance measures. Along with recall and precision, we were also interested in true and false negative rates, as these matrices are of great importance in surveillance and occupancy analysis applications, where an event of negative detection is as important as an event as positive detection. The F1 scores of all the combinations are provided in Table 4. Recall, precision, TNR, and FNR are illustrated in Figure 3. Here, we calculated our measures, i.e., F1 score, recall, precision, TNR, and FNR, as:

(1) $$F1\;\mathrm{score}=\frac{\mathrm{Precision}*\mathrm{Recall}}{\mathrm{Precision}+\mathrm{Recall}},\;\;\mathrm{Recall}=\frac{\mathrm{TP}}{\mathrm{TP}+\mathrm{FN}},\;\;\mathrm{Precision}=\frac{\mathrm{TP}}{\mathrm{TP}+\mathrm{FP}}$$

(2) $$\mathrm{TNR}=\frac{\mathrm{TN}}{\mathrm{TN}+\mathrm{FN}},\;\;\mathrm{FNR}=\frac{\mathrm{FN}}{\mathrm{FN}+\mathrm{TP}}$$

True Positives (TP) were defined as the number of persons that were correctly detected as persons and True Negatives (TN) as the number of images with zero persons correctly identified as having zero persons. False Positives (FP) represented the regions in the image with no person, but there was nonetheless a person detected. False Negatives (FN) represented the regions where persons were present, but the detector failed to recognize them.

Results presented in Table 4 indicated that for Combinations 2 to 5, when only one category was added at a time, viewpoint images significantly increased the value of the F1 score, indicated by green, while the images with the heat effect had the least impact on the results, indicated by red. For Combinations 6 to 11, the alliance of heat and weather effects and the alliance of viewpoint and image artifacts seemed to have the lowest performance. The combinations of heat effect and image artifacts and the combination of viewpoint and weather effects had the highest performance in terms of F1 score. For the last combinations, 12–15, we could see that including all categories exclusive of the weather effect had the highest F1 score of 89.74%, while the other combinations performed almost equally. The last combination with all data included as expected showed the maximum performance in terms of F1 score.

In looking individually at the results of each combinations, one noticeable observation was found with Combinations 2, 7 and 10. These combinations almost had the same performance. Although, if we looked at the number of images in Combinations 2 and 10, Combination 10 had more than three times the number of images as Combination 2. The same pattern could be observed in Combinations 12 and 16. The weather effect contained more than half of the data, but its inclusion increased the performance only by 1%.

The overall contribution of each category is also shown in the last row of Table 4. The mean was computed by taking the mean of all F1 scores in which a particular category was included. Results were consistent with the precision and TNR results, and heat effects had the lowest F1 score. The highest F1 score was obtained for the viewpoint category, which had images with good contrast and both far and close views. Moreover, this category introduced scene adaptation from an indoor to outdoor field environment. it could also be observed that although the image artifacts category had eight times fewer training images than weather effects, it had a better mean F1 score.

The results obtained from the experiment are also presented in Figure 3. Precision and recall are shown in Figure 3a, and FNR and TNR are shown in Figure 3b. it can be seen that for certain combinations, i.e., 3, 6, 10, and 13, there were visible dips in the precision and TNR values. The magnitude of the dip in precision was less than the TNR because only FP was considered in the calculation of precision, whereas in the TNR calculation, both FP and TN played a role.

If we looked at all these combinations, the common category was “heat effects”. The other noticeable effect was the decrease in the dip magnitude with the addition of more categories. As more and more categories were added to “heat effects”, the precision and TNR both improved. There was no significant change observed in the FNR results. However, the recall had an opposite effect from the precision and TNR, as the addition of the “heat effects” category improved recall. The details of this improvement are explained later in the section.

The precision and TNR were maximum for the image artifacts and weather effects categories. This was because occlusion and low resolution images were present in the image artifacts category, and the FP and FN reduced; whereas for weather effects, more images of empty fields with snow and shadow were added in the training data. Snow and shadow could sometimes resemble humans and be detected as persons. Therefore, with the addition of the weather effect category, FNR and TNR both improved.

Herrmann et al. concluded that an inverted thermal dataset had a resemblance to the grayscale of RGB data. Therefore, the domain adaptation was quicker when pretrained RGB weights were used. In our results, we could also observe a similar response in terms of recall.

We can see in Figure 3a that every time the heat effects category was added, recall improved. However, at the same time, precision and TNR reduced. All the other categories in Table 2, except heat effects, had images with persons in the dark background. Therefore, the heat effect category, which was 8% of the complete training dataset, acted as noise. In particular, similar temperature images had the most effect on reducing TNR. Any lesser contrast noise could be detected as FP. This problem could be solved by generalizing the dataset in a single domain by detecting the heat category events. Results also suggested that converting the whole dataset into inverted thermal images might be more beneficial, as this would help improve the recall and model adaptation.

**Figure 4:**
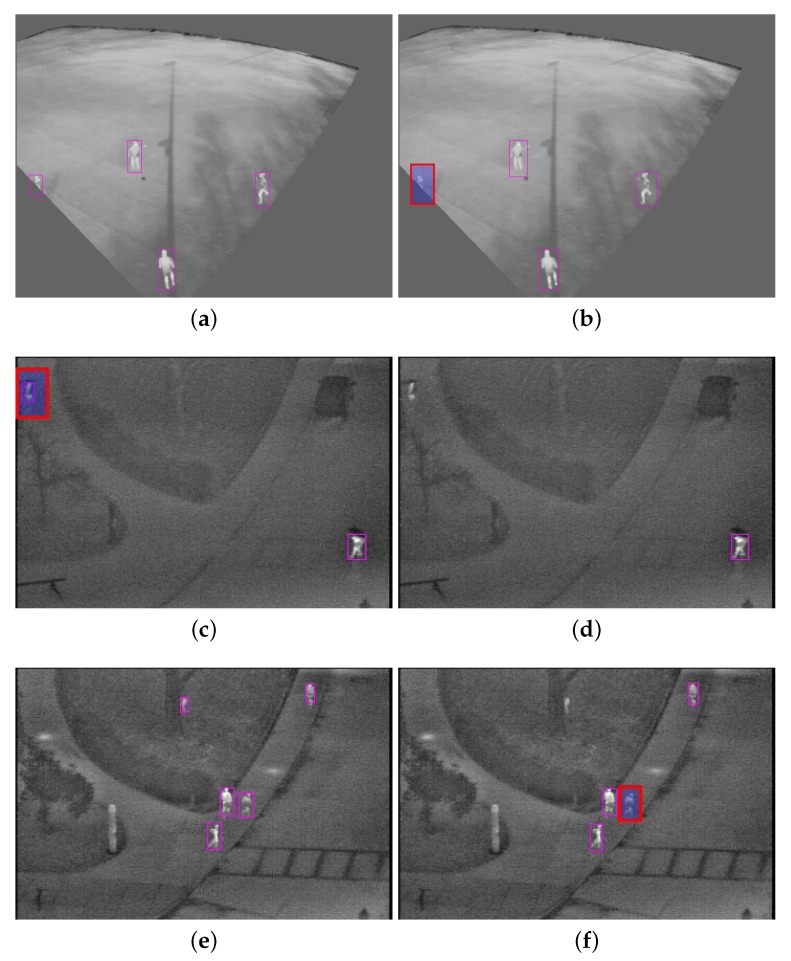
Example images for qualitative assessment. (**a**,**c**,**e**) are the results for Combination 16, while (**b**,**d**,**f**) are the results for the same images from Combination 12. The images (**a**,**b**) are from our test data, and the images (**c**–**f**) are from OSU-T [18]. In these images, highlighted red boxes are incorrect detections.

To select which category to include in training, it still depended on the target application. For example, if we compared Combinations 12 and 16, the increase in the F1 score was only 0.49% by including the data from the weather effect category. To show the effect of including the weather effect data, a few test images are shown in Figure 4. Figure 4a,b is from our dataset, and Figure 4c–d were taken from the publicly available CVC-09 database. Figure 4a,c,e was tested with Combination 16, where the weather effect was included; whereas Figure 4b,d,f is the results of the same images when the weather effect was not included, i.e., Combination 12. it can be seen that without the weather effect, TN and FN were better; however, with its inclusion, TP improved, but the FPR also increased. For example, if we needed the system for surveillance, then it would be important to avoid an FN event. In such cases, weather effects data would be required for training. Occupancy analysis has similar requirements.

### 4.3. Results on Publicly Available Datasets

We picked three public datasets to test the generalization of our trained weights for person detection. The datasets consisted of three different diverse datasets from Table 1: CVC-09 [26], OSU-T [18], and BU-TIV-atrium [46].

OSU-T was recorded outdoors with different weather conditions, as mentioned in Table 1. it consisted of 284 images. The data were captured from a far top viewpoint. CVC-09 was recorded from a camera in a car while driving. The images were divided into two subsets for day and night. CVC-09 (day) consisted of 2881 test images and 4223 training images, out of which 1112 were negative frames and 3111 positive frames. CVC-09 (night) consisted of 2883 test images and 3200 training images, out of which 1001 were negative frames and 2199 positive frames. BU-TIV was recorded indoors with a near top viewpoint. it had three sequences of videos with Views 1, 2, and 3. we chose its View 1 for our tests, which consisted of 3482 images.

Tests on publicly available datasets were performed in two sessions. Firstly the images were tested using the weights obtained from Combination 16, shown in Table 3. In the second session, tests were performed by adding 5% of the data from the public dataset to the Combination 16 dataset and retraining it.

For training the second session test, from OSU-T and BU-TIV, we added 5% of the whole data in training corresponding to 14 and 174 images, respectively, and from CVC-09 (day and night), 5% of the training data was added to the training set corresponding to 211 and 160 images, respectively. The number of iterations for learning was 100 to avoid overfitting due to a small number of training images.

**Figure 5:**
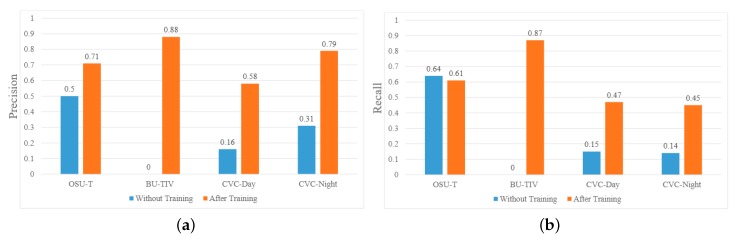
(**a**) Precision and (**b**) recall measures of different training weights on publicly available datasets. Here, the blue bars are the results tested by our thermal training weights, and orange bars are the results tested by our thermal training weights and further training by adding only 5% of the new dataset for 100 iterations.

Results of this experiment are presented in Figure 5. Blue bars are the results obtained from Combination 16 weights, and red bars are the results obtained after retraining Combination 16 with 5% of the public dataset. it can be seen that by using the weights from Combination 16, the performance was not good, and in the case of BU-TIV, the algorithm failed to detect anything. In BU-TIV, the viewpoint was different, and people appeared larger than in our dataset. However, with only 5% of training data and with 100 iterations, a significant increase in precision could be seen. The highest precision was obtained for BU-TIV and the lowest for the CVC data, with an average precision of 0.69%. In BU-TIV and OSU-T, there were no other heated objects present other than humans, and in OSU-T, the viewpoint was very similar to our dataset; therefore, good precision results were achieved.

**Figure 6:**
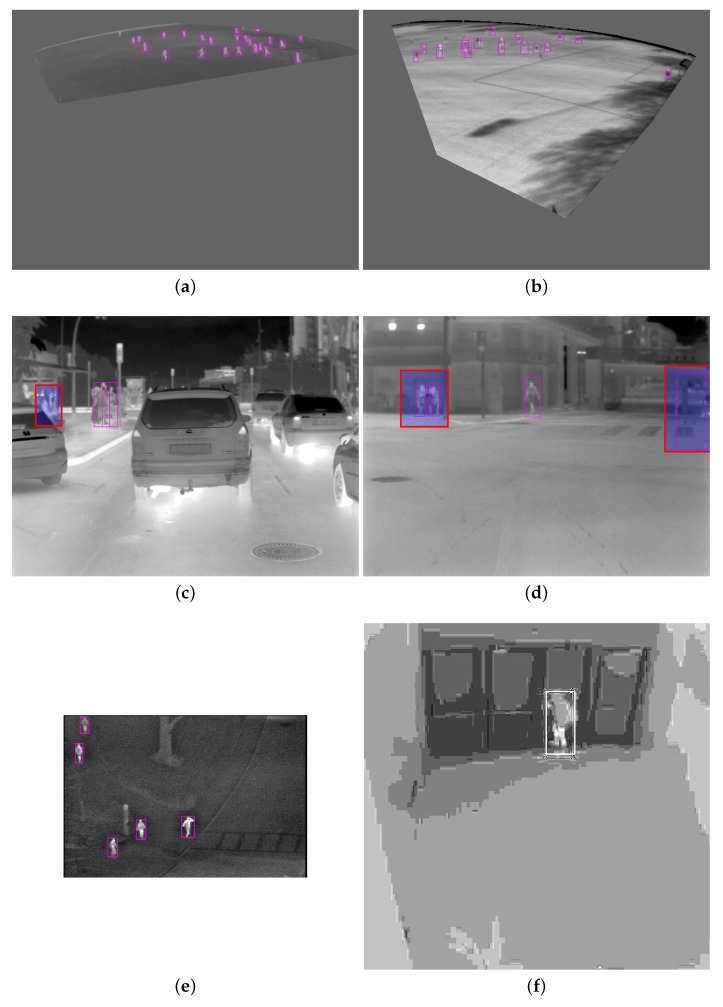
Example images for qualitative assessment. The images (**a**) and (**b**) are from our test data. The results of images (**a**,**b**) are obtained from Combination 16, shown in Table 4. Image (**c**) is from CVC-day [26], image (**d**) from CVC-night [26], image (**e**) from OSU-T [18], and image (**f**) from BU-TIV-atrium [46]. The contrast of (**f**) is adjusted for better visualization. In these images, highlighted red boxes are wrong detections.

In the CVC dataset, a significant difference between day and night results was observed. During the day, the temperatures of car bodies, tires, and other objects increased. Their pattern became similar to human body features, which increased FPR and decreased precision. Example results from all datasets used for evaluations are shown in Figure 6.

## 5. Conclusions

In this work, we reviewed publicly available thermal datasets that could be used for person detection, and we documented the lack of diversity in these datasets. we also studied and presented a new thermal dataset and found nine different phenomena that could occur in outdoor soccer fields. The phenomena were further categorized into four categories. The impact of each category was studied for model generalization using transfer learning. Results showed that each category benefited the model generalization differently. The results showed that depending on the application, categories could be selected intelligently to obtain the desired results. The weights obtained from our dataset were further tested on three publicly available datasets. For a relatively small amount of training data from a new domain and with few iterations, good performance was achieved for person detection. Results showed that our weights could be used for model adaptation for a new domain. This will help researchers save the effort of annotating large datasets and also the time for training a new network from scratch. Moreover, with weights for YOLOv3, our new dataset is made publicly available for further research. 

## Figures and Tables

**Table 1:** Available thermal datasets for person detection and the characteristics of each dataset. “Application area/main scene characteristics” summarizes the main features of the videos in each dataset. “Viewpoint” is estimated by generally looking at the image for the camera angle and the distance of persons from the camera.

Name	# of Frames	# of Sequences	Viewpoint	Application Area/ Main Scene Characteristics	Camera/Image Specifications
KAIST [1]	95 k		Near front	outdoor traffic, day and night multispectral	640 × 480, 20 Hz
OSU Color Thermal (CT) [20]	17 k		Far top	Outdoor walkway	Raytheon PalmIR 250D, 320 × 240, 30 Hz
AAU-VAP TPD [41]	5.7 k	3	Near front	Indoor office	Axis Q1922 640 × 480 30 Hz
LITIV-VAP [22]	4.3 k		Near front	Indoor hall	
CVC-09 [26]	11 k		Near	Traffic pedestrian, day and night	640 × 480
CVC-14 [42]	7.7 k		Near	Traffic pedestrian, day and night	
LITIV-2018 [43]		3	Near front	Indoor hall	
OSU Thermal (T) [18]	0.2 k		Far top	Outdoor pedestrian haze, fair, light rain, partially cloudy	Raytheon 300D, 320 × 240, 30 Hz
ASL-TID [44]	4.3 k	8	Varied	Outdoor varied background, person, cat, horse	FLIR Tau 324 × 256
Terravic Motion IR [32]	23.7 k	18	Varied	Outdoor tracking, surveillance, indoor hallway, plane tracking, underwater and near-surface motion, background motion	Raytheon L-3 Thermal-eye 2000AS, 320 × 240
LSI Dataset [45]	15.2 k	13		Outdoor pedestrian Hz	Intigo Omega imager, 164 × 129
BU-TIV [46] Benchmark Atrium	7.9 k	2	Near	Indoor atrium	512 × 512
Lab	26.7 k	3	Near	Indoor and	512 × 512
Marathon	1 k		Very far	outdoor marathon	1024 × 640
VOT-TIR 2015 [47] Birds	270	1	Near front	Fair outdoor	640 × 480, 30 Hz
Crossing	301	1	Near top	Fair outdoor	640 × 480, 30 Hz
Crouching	618	1	Near front	Outdoor roadside	640 × 480, 30 Hz
Crowd	71	1	Near front	Outdoor roadside occluded	640 × 512, 30 Hz
Street	172	1	Far front	Outdoor street	640 × 480, 30 Hz
Saturated	218	1	Near front	Outdoor street occluded	640 × 480, 30 Hz
Mixed distractor	270	1	Near front	Indoor	527 × 422, 30 Hz
Hiding	358	1	Near front	Indoor	263 × 210, 30 Hz
Garden	676	1	Near top	Outdoor garden	324 × 256, 30 Hz
Depth-wise crossing	851	1	Medium top	Outdoor fair roadside	640 × 480, 30 Hz
Trees	665	1	Far top	Outdoor dark	640 × 480, 30 Hz
Thermal soccer dataset [40]	3000	4	Near top	Indoor soccer arena	640 × 480, 30 Hz

**Table 2:** Key characteristics of the proposed training data.

Category	Phenomena	# of Frames	# of Persons
Viewpoint	Good condition	122	632
	Far viewpoint	64	652
Heat effects	Opposite temperature	72	792
	Similar temperature	107	644
Image artifacts	Low resolution	158	734
	fOcclusion	20	305
Weather effects	Shadow	171	742
	Snow	1060	168
	Wind	167	921

**Table 3:** List of combinations for tests.

#	Combinations	#	Combinations
1.	Indoor	9.	Indoor+heat effects+image artifacts
2.	Indoor+viewpoint	10.	Indoor+heat effects+weather effects
3.	Indoor+heat effects	11.	Indoor+image artifacts+weather effects
4.	Indoor+image artifacts	12.	Indoor+Viewpoint+heat effects+image artifacts
5.	Indoor+weather effects	13.	Indoor+viewpoint+heat effects+weather effects
6.	Indoor+viewpoint+heat effects	14.	Indoor+heat effects+image artifacts+weather effects
7.	Indoor+viewpoint+image artifacts	15.	Indoor+viewpoint+image artifacts+weather effects
8.	Indoor+viewpoint+weather effects	16.	Indoor+viewpoint+heat effects+image artifacts+weather effects

**Table 4:** F1 score of combinations. Here, **X** indicates the category added in a combination. Other than indoor data, Combinations 2–5 only had one category of images, Combinations 6–11 two categories of images, and Combinations 12–15 three categories of images. Lastly, Combination 16 had all categories. The red color shows the worst-performing combinations, and the green color shows the best performing combinations within each section.

Combinations	Indoor	Viewpoint	Heat Effects	Image Artifacts	Weather Effects	F1 Score
**1**	X					63.35
**2**	X	X				81.52
**3**	X		X			75.35
**4**	X			X		78.39
**5**	X				X	79.68
**6**	X	X	X			83.63
**7**	X	X		X		81.74
**8**	X	X			X	87.37
**9**	X		X	X		88.24
**10**	X		X		X	81.71
**11**	X			X	X	83.04
**12**	X	X	X	X		**89.74**
**13**	X	X	X		X	87.57
**14**	X		X	X	X	87.34
**15**	X	X		X	X	86.99
**16**	X	X	X	X	X	**90.23**
Mean	82.87	86.10	85.48	85.78	85.49	
